# Comparison of the efficacy of steroid-free versus classic steroid-containing regimens in primary membranous nephropathy

**DOI:** 10.3389/fphar.2024.1286422

**Published:** 2024-02-14

**Authors:** Hui-Lin Xing, Dong-Hong Ma, Jin Li, Qing-Yu Xu, Li-Ke Ji, Qiong-Jie Zhu, Yu-Qing Luo, Ming-Hao Guo

**Affiliations:** Department of Nephrology, Kidney Disease Hospital, The First Affiliated Hospital of Xinxiang Medical University, Xinxiang, Henan, China

**Keywords:** rituximab, tacrolimus, prednisone, primary membranous nephropathy, steroids

## Abstract

**Objective:** To compare the efficacy of a steroid-free regimen with steroid-based treatment in managing primary membranous nephropathy (PMN) and investigate the potential benefits of steroid-free regimens in PMN therapy.

**Methods:** This was a single-centre prospective cohort study. A total of 81 patients were divided into two groups according to their medication regimen: a rituximab (RTX)/tacrolimus (TAC) group (low-dose RTX combined with low-dose TAC group, without steroids, n = 31) and a prednisone (P)/TAC group (P combined with TAC group, n = 61). The changes in 24-h urine protein quantification, levels of blood albumin, blood creatinine, total cholesterol, triglyceride and fasting blood glucose as well as anti-phospholipase A2 receptor antibody titres were observed in both groups before treatment and after 1, 3, 6 and 12 months of treatment. Clinical remission (complete and partial remission), serological remission and recurrence were assessed in both groups after treatment, and the occurrence of adverse reactions was observed.

**Results:** 1) Before treatment, there was no significant difference in baseline values between the two groups (*p* > 0.05). 2) After 12 months of treatment, the 24-h proteinuria and total cholesterol levels in the RTX/TAC group were significantly lower than those in the P/TAC group (*p* < 0.05). 3) After 6 months of treatment, the clinical remission rate of the RTX/TAC group was significantly higher than that of the P/TAC group (*p* < 0.05). After 12 months of treatment, the clinical remission rate of the RTX/TAC group was significantly higher than that of the P/TAC group (*p* < 0.05). (4) After 3, 6 and 12 months of treatment, serological remission rates in the RTX/TAC group were significantly higher than those in the P/TAC group (*p* < 0.05). During treatment, the anti-PLA2R antibody titres in the RTX/TAC group remained lower than those in the P/TAC group (*p* < 0.05).

**Conclusion:** The low-dose RTX combined with low-dose TAC steroid-free regimen induces serological remission in patients with PMN earlier than the classic regimen of P combined with TAC, and there was no significant difference in adverse effects between the two groups. Besides, the long-term clinical remission effect of low-dose RTX combined with low-dose TAC is better than that of P combined with TAC.

## Introduction

The 2021 Kidney Disease Improving Global Outcomes (KDIGO) clinical practice guidelines recommend immunosuppressive therapy for patients with primary membranous nephropathy (PMN) who have been evaluated as being at risk of progression through clinical observation. This therapy uses rituximab (RTX), steroids combined with cyclophosphamide, or calcineurin inhibitors (CNI) such as tacrolimus (TAC) (Rovin et al., 2021). Rituximab is one of the first-line drugs recommended in the current guidelines for the treatment of PMN, but its effect as a single drug treatment is slow. Prednisone (P) combined with TAC is also a first-line scheme for the treatment of PMN, but the side effects of the long-term use of P are evident, with most patients having steroid restrictions, whereas the long-term use of TAC has some shortcomings such as prominent nephrotoxicity and a high recurrence rate. Some studies have suggested that ultra-low-dose RTX combined with low-dose TAC is more effective and safer in the treatment of refractory PMN than TAC alone, suggesting the potential value of this combination therapy (Pathak et al., 2020; Zhu et al., 2021). Although the guidelines suggest treatment options for PMN, there are still clinical controversies about the use of RTX in combination with other immunosuppressive regimens. There is insufficient clinical evidence to assess whether RTX in combination with other immunosuppressive agents can improve the efficacy of PMN treatment, it has a better safety profile than traditional hormonal regimens or it is more suitable for the treatment of patients with PMN in China.

By comparing the clinical remission (primary outcome) and safety of steroid-free regimens and traditional steroid regimens, this study attempts to provide a theoretical basis for the formulation of an improved treatment plan for patients with PMN and a new option for the clinical treatment of PMN.

## Materials and methods

### Patients

Adult patients with PMN treated in the Kidney Disease Hospital, First Affiliated Hospital of Xinxiang Medical University, between August 2021 and February 2022 were selected through simple random sampling. Treatment plan economic factors were comprehensively evaluated, and the patients’ subjective choice of drugs was respected. The included patients were treated with either a steroid-free regimen (low-dose RTX combined with low-dose TAC) or classic regimen (P combined with TAC). Inclusion criteria: (i) biopsy-proved PMN, (ii) 8.0 g d^−1^ > 24-h proteinuria >4.0 g d^−1^, (iii) estimated glomerular filtration rate (eGFR) > 30 mL·min^−1^·1.73 m^−2^. Exclusion criteria: (i) patients with membranous nephropathy secondary to other causes, tested positive for hepatitis B or HIV, had malignancy or autoimmune diseases; (ii) pregnant or breastfeeding women; (iii) patients previously treated with immunosuppressive therapy; (iv) patients with severe infection, cardiac insufficiency or other serious complications (Scolari et al., 2019; Scolari et al., 2021). Participants were withdrawn from the study if they had elevated serum creatinine and eGFR <30 mL·min^−1^·1.73 m^−2^ during treatment, spontaneous drug withdrawal leading to disease recurrence or were lost to follow-up. The patients included in this study met the moderate-risk criteria outlined in the 2021 KDIGO guidelines. The clinical remission rate of the participants was the main outcome index.

According to the preliminary experimental results of this study, after 3 months of treatment, the clinical remission rate of the RTX/TAC group was 29% and that of the P/TAC group was 58%. According to the sample size formula

$$n=\frac{2\overset{-}{p}\overset{-}{q}{\left(Z_{\alpha }+Z_{\beta }\right)}^{2}}{{\left(P1-P2\right)}^{2}},$$

if the bilateral α = 0.05 was set with 90% confidence, a total of 92 patients should be included in the study. Based on a sample size ratio of 1:2 (RTX/TAC group to P/TAC group), the patients were divided into groups using the random number table method. Finally, 31 patients in the RTX/TAC group and 61 in the P/TAC group were enrolled in the study. All patients provided written informed consent. The study was approved by the ethics committee of the First Affiliated Hospital of Xinxiang Medical University (No. 2020001).

### Interventions

RTX/TAC group: Low-dose RTX was defined as an infusion dosage of 375 mg·m^−2^ on days 1 and 15, and B-cell depletion was defined as <5 B cells·µl^−1^. If circulating B cells >5 B cells·µl^−1^, an additional dosage of 200 mg of RTX was applied to maintain the circulating B-cell depletion. Low-dose TAC was administered at 0.05–0.075 mg·kg^−1^·d^−1^ orally, given at 12-h intervals and adjusted to a serum concentration level of 3–5 ng·mL^−1^ for 12 months. P/TAC group: A P starting dose of 0.5 mg·kg^−1^·d^−1^ (Ramachandran et al., 2016; Zou et al., 2019) orally. During the follow-up period, the dosage was gradually reduced to discontinuation depending on the patient’s response, and the treatment was maintained for 12 months. TAC was administered at an initial dose of 0.05–0.10 mg·kg^−1^·d^−1^ orally, given at 12-h intervals and adjusted to a serum concentration level of 4–10 ng·mL^−1^ for 12 months. The TAC concentration level was adjusted based on our centre’s treatment experience.

### Outcomes

(i) Primary outcomes: Complete remission: 24-h proteinuria <0.3 g·d^−1^, serum albumin >35 g·L^−1^ and stable renal function. Partial remission: 24-h proteinuria <3.5 g·d^−1^ and >50% reduction from baseline, serum albumin >30 g·L^−1^ and stable renal function. Clinical remission: Both complete remission and partial remission. Relapse: After complete or partial remission, 24-h proteinuria increases and serum albumin levels decrease. (ii) Secondary outcomes included changes in 24-h proteinuria, time for the anti-PLA2R antibody to turn negative, serological remission, levels of serum albumin, serum creatinine, eGFR, cholesterol, triglyceride and fasting blood glucose, as well as anti-PLA2R antibody titres and adverse events. Serological remission was defined as anti-PLA2R antibodies turning negative and anti-PLA2R antibody titres <14 RU·ml^−1^. All indexes were analysed before treatment and at 1, 3, 6 and 12 months after the end of treatment.

### Statistical analysis

Statistical analysis was performed using the statistical SPSS 26.0 software. All normally distributed continuous variables are expressed as mean ± standard deviation. Continuous variables with skewed distribution are expressed as a median (interquartile range). Categorical variables are expressed as percentages. The *t*-test was used to analyse normally distributed continuous variables from different groups. The Mann–Whitney *U* test was applied to continuous variables with skewed distribution. The Chi-square test or Fisher exact test was used to compare categorical variables. Survival curves were calculated using Kaplan–Meier estimates for survival distribution. The significance level was set at *P* value less than 0.05.

## Results

### Patients

The follow-up period was 12 months. By the end of follow-up, 3 patients in the RTX/TAC group had withdrawn from the treatment; 1 refused the study protocol after enrolment and withdrew from the study before treatment, and 2 experienced relapse following the self-discontinuation of TAC after achieving clinical remission, changed treatment regimen and withdrew from the study in the fourth month of treatment. In the P/TAC group, 4 patients were discharged from the study as a result of relapse caused by the self-discontinuation of TAC after achieving clinical remission; their treatment was replaced with different regimens at 4, 6 and 10 months. Another 4 patients in this group were lost to follow-up. However, 28 patients in the RTX/TAC group and 53 in the P/TAC group received complete treatment interventions.

### Baseline characteristics of the two groups

The mean age of the RTX/TAC group was 50.79 ± 12.15 years, with 21 men and 7 women. The mean age of the P/TAC group was 47.47 ± 12.67 years, with 37 men and 16 women. There were no significant differences in age, sex, blood pressure, levels of 24-h proteinuria, blood albumin, blood creatinine, total cholesterol, triglyceride and fasting glucose or anti-PLA2R antibody titres, anti-PLA2R antibody positive rates and the CD19^+^B lymphocyte count between the two groups (*p* > 0.05) (Table 1).

**TABLE 1:** Baseline characteristics of two groups.

Characteristic	RTX/TAC (n = 28)	P/TAC (n = 53)	*t/*χ^2^/*Z*	*p*-Value
Age, yr	50.79 ± 12.15	47.47 ± 12.67	1.135	0.260
Sex			0.240	0.622
Male, n (%)	21 (75.0)	37 (69.8)		
Female, n (%)	7 (25.0)	16 (30.2)		
Systolic, (mmHg)	138.86 ± 20.61	140.83 ± 19.13	−0.430	0.668
Diastolic, (mmHg)	86.79 ± 14.75	91.26 ± 12.36	−1.449	0.151
24 h urinary protein, (g·d^-1^)	6.40 ± 2.22	6.32 ± 2.21	0.156	0.877
Blood albumin, (g·L^-1^)	24.91 ± 4.42	26.67 ± 4.75	−1.615	0.110
Blood creatinine, (μmol·L^-1^)	65.02 ± 17.85	64.06 ± 15.58	−0.513	0.609
eGFR, (ml·min^-1^·1.73m^-2^)	127.23 ± 35.39	128.54 ± 40.06	−0.145	0.885
Total cholesterol, (mmol·L^-1^)	7.56 ± 2.41	7.88 ± 2.28	−0.592	0.556
Triglyceride, (mmol·L^-1^)	2.96 ± 1.80	2.03 ± 1.51	2.456	0.056
Fasting glucose, (mmol·L^-1^)	5.15 ± 0.64	5.20 ± 0.96	−0.265	0.792
Anti-PLA2R antibody titer, (RU·ml^-1^)	61.55 (19.98,140.83)	38.50 (7.20,120.10)	−1.200	0.230
Anti-PLA2R antibody			2.337	0.126
Anti-PLA2R–positive patients, (%)	23 (82.14)	35 (66.04)		
Anti-PLA2R–negative patients, (%)	5 (17.86)	18 (33.96)		
CD19^+^ B lymphocyte count, (B cells·ul^-1^)	402.96 ± 170.53	354.38 ± 209.84	1.054	0.295

### Changes in clinical remission in the two groups

**FIGURE 1:**
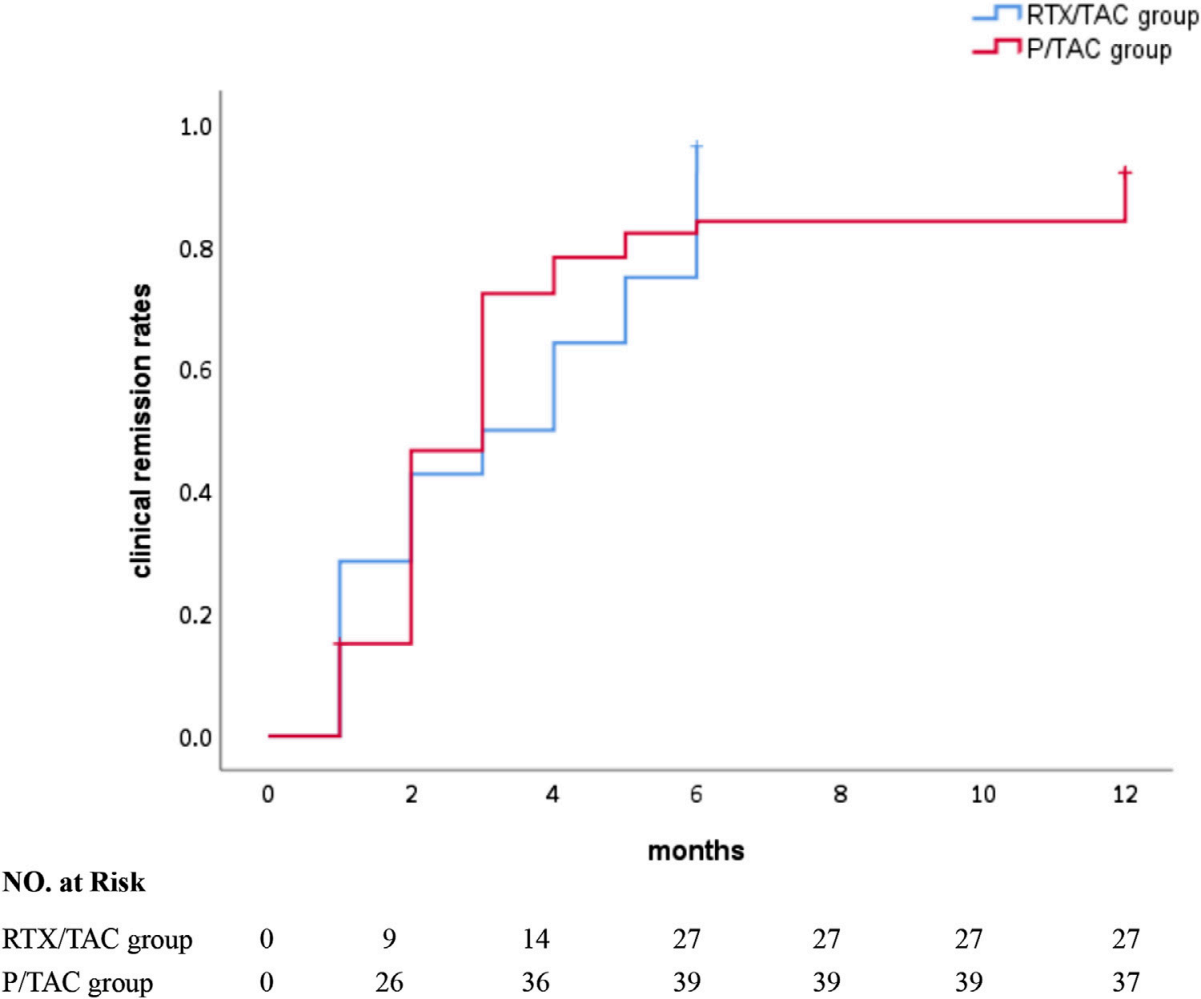
Clinical remission rates of the two groups.

After 1 month of treatment, the clinical remission rates in the RTX/TAC group were similar to those in the P/TAC group (28.6% vs 20.8%, *p* > 0.05). After 3 months of treatment, although the partial remission rate in the RTX/TAC group was lower than that in the P/TAC group (25.0% vs. 52.8%, *p* < 0.05), the clinical remission rates in the RTX/TAC group were similar to those in the P/TAC group (32.1% vs. 52.8%, *p* > 0.05). After 6 months of treatment, the clinical remission rates in the RTX/TAC group were significantly higher than those in the P/TAC group (96.4% vs. 73.6%, *p* < 0.05). After 12 months of treatment, the clinical remission rates in the RTX/TAC group were significantly higher than those in the P/TAC group (96.4% vs. 69.8%, *p* < 0.05). By the end of follow-up, no patients in the RTX/TAC group had relapsed, but 9 had relapsed in the P/TAC group; no difference was identified between the groups (*p* > 0.05) (Table 2). The median time to clinical response was 3.0 (1.0, 5.0) months for the RTX/TAC group and 3.0 (2.0, 4.0) months for the P/TAC group; the difference was statistically significant (*p* = 0.030) (Figure 1).

**TABLE 2:** The changes of clinical remission in two groups.

	n	Complete remission, (%)	Partial remission, (%)	Clinical remission (%)	Relapse, (%)
RTX/TAC group	28				
1 month		0	8 (28.6)	28.6	0
3 months		2 (7.1)	7 (25.0)^a^	32.1	0
6 months		5 (17.9)	22 (78.6)	96.4^a^	0
12 months		10 (35.7)	17 (60.7)	96.4^a^	0
P/TAC group	53				
1 month		0	11 (20.8)	20.8	0
3 months		0	28 (52.8)	52.8	0
6 months		9 (17.0)	30 (56.6)	73.6	0
12 months		10 (18.9)	27 (50.9)	69.8	9 (17.0)

Compared with the P/TAC, group.

^a^ *p* < 0.05 at the same time point.

### Changes in anti-PLA2R antibodies and serological remission in the two groups

In the RTX/TAC group, anti-PLA2R antibodies were significantly decreased compared with before treatment (*p* < 0.01). The median time for anti-PLA2R antibodies to turn negative was 2.00 (1.00–3.50) months in the RTX/TAC group and 6.00 (3.00–6.00) months in the P/TAC group; the difference was statistically significant (*p* < 0.01). The serological remission rates in the RTX/TAC group were significantly higher than those in the P/TAC group (*p* < 0.01) (Table 3).

**TABLE 3:** The changes of anti-PLA2R antibody and serological remission in two groups.

	n	Anti-PLA2R antibody	Anti-PLA2R–positive patients, (%)	Serological remission, (%)
RTX/TAC group	28			
Baseline		61.55 (19.98, 140.83)	23 (82.14)	
3 months		2.90 (2.05, 5.50)^a^	5 (17.9)	18 (78.3)^a^
6 months		2.40 (1.35, 3.65)^a^	1 (3.6)	22 (95.7)^a^
12 months		2.15 (1.33, 2.80)^a^	1 (3.6)	22 (95.7)^a^
P/TAC group	53			
Baseline		38.50 (7.20, 120.10)	35 (66.0)	
3 months		25.90 (9.90, 84.60)	31 (58.5)	4 (11.4)
6 months		5.00 (1.80, 22.13)	23 (43.4)	12 (34.3)
12 months		17.00 (4.35, 53.10)	27 (50.9)	8 (22.9)

Compared with the P/TAC, group.

^a^ *p* < 0.05 at the same time point.

### Changes in laboratory indexes in the two groups

After 12 months of treatment, 24-h proteinuria levels in both groups were significantly decreased compared with baseline, and 24-h proteinuria levels in the RTX/TAC group were lower than those in the P/TAC group (*p* < 0.05). After 12 months of treatment, the serum albumin level of both groups was significantly increased compared with baseline (*p* < 0.05), but there was no significant difference between the groups (*p* > 0.05). After 12 months of treatment, the total cholesterol level of the RTX/TAC group was lower than that of the P/TAC group. By the end of follow-up, the levels of eGFR, triglyceride, fasting blood glucose and serum creatinine in the two groups were similar (*p* > 0.05) and also similar to those before treatment (*p* > 0.05) (Table 4).

**TABLE 4:** The changes of laboratory indexes in two groups.

Characteristic	RTX/TAC group (n = 28)		P/TAC group (n = 53)	
	Baseline	12 months	Baseline	12 months
24 h urinary protein, (g·d^-1^)	6.40 ± 2.22	0.65 ± 0.52^ab^	6.32 ± 2.21	2.24 ± 2.65^b^
Serum albumin, (g·L^-1^)	24.91 ± 4.42	39.72 ± 2.98^b^	26.67 ± 4.75	37.23 ± 6.57^b^
Serum creatinine, (μmol·L^-1^)	65.02 ± 17.85	69.61 ± 14.39	64.06 ± 15.58	70.43 ± 18.86
eGFR/(ml·min^-1^·1.73m^-2^)	127.23 ± 35.39	111.72 ± 34.05	128.54 ± 40.06	118.94 ± 44.73
Total cholesterol, (mmol·L^-1^)	7.56 ± 2.41	4.92 ± 1.07^a^	7.88 ± 2.28	5.51 ± 1.26
Triglyceride, (mmol·L^-1^)	2.96 ± 1.80	2.03 ± 0.93	2.03 ± 1.51	1.99 ± 0.90
Fasting glucose, (mmol·L^-1^)	5.15 ± 0.64	5.88 ± 0.93	5.20 ± 0.96	6.13 ± 1.59

Compared with the P/TAC, group.

^a^ *p* < 0.05 at the same time point.

^b^ *p* < 0.05 compared with this group in baseline.

### Adverse effects

Two patients in the RTX/TAC group developed infusion reactions (7.1%); 1 developed fever on the first day of RTX infusion, and the other developed chest discomfort on the first day of RTX infusion (none of these 2 patients had the same type of infusion reaction on the second RTX infusion). Two patients in the P/TAC group developed pulmonary infection during treatment (3.8%). No definite adverse effects were observed in the remaining patients.

## Discussion

In this prospective study, we compared the efficacy and adverse effects of treatment in RTX/TAC group with that of P/TAC group. The results showed that a low dose of RTX combined with a low dose of TAC induced the serological remission of PMN earlier than P combined with TAC, and continuing long-term clinical remission was significantly improved compared with P combined with TAC. No serious adverse effects occurred in either group.

Primary membranous nephropathy is an autoimmune disease in which auto-reactive B cells produce autoantibodies, interact with T cells and release cytokines to promote the development of PMN. Rituximab is a monoclonal antibody against the B-cell CD20 antigen, which can effectively remove B cells and block the production of disease-causing antibodies through antibody- or complement-dependent cytotoxic effects and the induction of apoptosis (Salles et al., 2017). However, TAC is a CNI and reduces proteinuria production by inhibiting T-cell activation and reducing the expression of various cytokines such as interleukin-2 to reduce the immune inflammatory response (Rauch et al., 2009). Therefore, RTX combined with TAC therapy can target the removal of B cells and T cells, thus suppressing abnormal immune responses, theoretically offering a reasonable treatment option for PMN. The STARMEN study (Fernández-Juárez et al., 2021) compared the efficacy of sequential TAC combined with RTX and cyclophosphamide combined with corticosteroids in the treatment of PMN, but whether sequential RTX/TAC therapy was significantly more effective than treatment with cyclophosphamide combined with corticosteroids was unclear. In the present study, the clinical remission rate at 12 months of treatment was significantly higher in the RTX/TAC group than in the P/TAC group, and although no patients experienced relapse in the RTX/TAC group, 9 experienced relapse in the P/TAC group. The objective of RTX/TAC sequential therapy is to enhance the immunosuppressive effect of TAC with RTX and prevent PMN recurrence when TAC is reduced or discontinued. The STARMEN study did not achieve the expected therapeutic purpose. The main difference between our study and the STARMEN study is that the timing of the combination of RTX and TAC was different; that is, TAC and RTX were administered sequentially in the STARMEN study, whereas RTX and TAC were administered simultaneously in this study.

In this study, the clinical remission rate after 6 months of treatment was 96.4% in the RTX/TAC group compared with 73.1% reported in another study (Zhu et al., 2021). This difference may be attributed to the inclusion of newly treated patients with PMN in our study, whereas the other study focused on patients with refractory PMN. The median response time in the RTX/TAC group was 3.00 months, which was consistent with the median response time in the P/TAC group. Although the present study did not confirm that the response time of the RTX/TAC group was earlier than that of the P/TAC group, based on previous clinical experience, the clinical response time of the RTX/TAC group in the present study was earlier than that of RTX monotherapy. This may be because of the dual inhibition effect of combined therapy on B cells and T cells, accelerating the time to remission in patients with PMN.

Anti-PLA2R antibodies have been identified as a key biomarker of PMN pathogenesis, and RTX can effectively inhibit anti-PLA2R antibody production (Beck et al., 2011; Gao et al., 2021). In this study, after 3 months of treatment, 18 patients in the RTX/TAC group achieved serological remission, whereas 4 patients in the P/TAC group achieved serological remission. After 6 months of treatment, 22 patients in the RTX/TAC group achieved serological remission and only 1 patient did not; in the P/TAC group, 12 patients achieved serological remission and 23 patients did not. After 12 months of treatment, 1 patient in the RTX/TAC group and 27 in the P/TAC group had no serological response. Comparing the anti-PLA2R antibody titres of the two groups, those of the RTX/TAC group were lower than those of the P/TAC group during treatment; that is, the decrease in the RTX/TAC group was significantly higher than that in the P/TAC group. Consistent with the GEMRITUX and MENTOR studies, the serological remission rate of patients in the RTX/TAC group in this study was higher than that in the P/TAC group, and the decline rate of anti-PLA2R antibodies was faster and more substantial, which was considered to be related to the targeted clearance of RTX on PLA2R antibodies in combination therapy (Dahan et al., 2017; Ponticelli and Moroni, 2019).

In the present study, it was found that the clinical response rates were 32.1%, 96.4% and 96.4% in the RTX/TAC group after 3, 6 and 12 months of treatment, respectively, and the serological response rates were 78.3%, 95.7% and 95.7%, respectively; that is, the serological response rates in the RTX/TAC group were earlier than the clinical response. This is consistent with the findings of most studies. Due to the slow remodelling of the podocyte structure and slow repair of the glomerular filtration barrier structure and function, albuminuria takes time to decline. However, RTX rapidly reduces the production of anti-PLA2R antibodies through the targeted clearance of B cells, producing a time gap between serological remission and the decline in albuminuria (Ponticelli and Moroni, 2019). The onset of albuminuria comes weeks or months later than antibody production, and the decline in albuminuria comes weeks or months later than the point at which antibodies turn negative. The time gap between serological response and clinical response reflects the slower remodelling of the podocyte structure and repair of the glomerular filtration barrier structure and function, resulting in a longer period of decline in albuminuria (Francis et al., 2016). Therefore, whether 24-h proteinuria levels can be used as a clinical biomarker to evaluate the efficacy of immunosuppressive therapy remains controversial.

The optimal regimen of RTX for PMN has not yet been standardised. One study proposed ultra-low-dose RTX (100 mg initially, and an additional 100 mg when CD19^+^ B cells >5 B cells·µl^−1^) in combination with low-dose TAC (starting at 0.025 mg·kg^−1^·d^−1^) for refractory PMN and compared with TAC monotherapy (starting at 0.050 mg·kg^−1^·d^−1^) (Ponticelli and Moroni, 2019). The results showed that the clinical remission rates were significantly higher in the RTX/TAC group than in the TAC group after 6 and 12 months of treatment; the incidence of adverse events was significantly lower in the RTX/TAC group than in the TAC group after 12 months of treatment. The combination of ultra-low-dose RTX and low-dose TAC is more effective and safer than TAC monotherapy; therefore, RTX/TAC combination therapy may be a potential option for patients with refractory PMN. Previous studies focused on patients with refractory PMN, but the participants in this study were patients with primary PMN, and further exploration is needed to determine whether smaller doses of RTX are needed for patients with primary PMN in the future. The standard four-dose regimen (375 mg·m^−2^ per week, intravenous infusion for 4 weeks) or two-dose regimen (infusion dosage of 1 g on days 1 and 15) is now commonly used in clinical practice to induce remission and then continue remission with additional RTX following the same regimen at month 6 or with an equal dose of RTX, depending on the B-cell count (if CD19^+^ B cells are >5 B cells·µl^−1^). The cumulative cost of RTX for 6 months of treatment in both of these regimens was significantly higher than that of the drugs in the RTX/TAC group in the present study. The RTX combined with the TAC treatment regimen proposed in this study was economically less costly and more acceptable to patients compared with the standard four-dose regimen and two-dose regimen.

In this study, the median time to the first addition of 200 mg of RTX in the RTX/TAC group was 4.0 (3.0, 5.0) months, and the pharmacokinetic profile of RTX showed that 1 mg·m^−2^ of RTX instantaneously cleared 97% of circulating B cells and 100 mg of RTX maintained B-cell depletion for 3 months (Schoergenhofer et al., 2018). The degree of B-cell depletion has been previously reported to be a key factor affecting efficacy; the addition of 200 mg of RTX to shorten the dosing interval in this study could therefore theoretically maintain B-cell depletion and thus achieve efficacy similar to that of the standard regimen.

In this study, 2 patients in the RTX/TAC group experienced mild infusion reactions, and 2 patients in the P/TAC group had lung infections. Considering that RTX can also weaken the patient’s immunity while clearing B lymphocytes, reducing the dose of RTX can reduce the occurrence of adverse reactions in patients. The fewer adverse reactions in the RTX/TAC group in this study could be related to the low dose of RTX. Because of the high recurrence rate when TAC is reduced or discontinued, most patients need long-term application. Long-term application can easily lead to abnormal glucose tolerance, and combined steroids can increase the risk of diabetes. In addition, elevated serum creatinine is also a common adverse reaction of TAC (Cui et al., 2017; Ramachandran et al., 2017; Lin et al., 2019; Shao et al., 2019). In this study, 2 patients in the RTX/TAC group withdrew from the study after the self-discontinuance of TAC following partial remission and relapse after 3 months of treatment. No increase in blood glucose was found in the two groups, which was considered to be related to the control of TAC blood concentrations within the target range. In this study, the levels of serum creatinine and eGFR were stable in both groups, which was considered to be related to the recovery of renal function after regular TAC treatment. The effect of TAC on renal function may be affected by drug dose and blood concentration. Although TAC in both the RTX/TAC and P/TAC groups in this study was within the target blood concentration range, clinicians should still pay attention to monitoring changes in renal function to avoid renal injury or the progression of renal insufficiency. As this is a 12-month clinical study, long-term follow-up will be required to further evaluate adverse effects in both groups.

This study clarified that the long-term clinical remission level in the RTX/TAC group was better than that in the P/TAC group and also provided a new choice for patients with steroid contraindications, steroid resistance and steroid rejection as well as patients with PMN who should not use steroids due to complications such as diabetes. Moreover, during TAC treatment, RTX was added according to the CD19+B-cell count, which could effectively maintain remission and avoid relapse caused by TAC reduction or drug withdrawal. Since this study is a single-centre prospective small-sample cohort study, limited by the number of participants and short follow-up time, the long-term efficacy and safety of low-dose RTX combined with low-dose TAC in the treatment of PMN still need to be further evaluated.

## Data Availability

The original contributions presented in the study are included in the article/Supplementary Material, further inquiries can be directed to the corresponding author.
